# Defining treatment success in children with surgical conditions

**DOI:** 10.1136/archdischild-2023-326156

**Published:** 2023-12-22

**Authors:** Oliver Rivero-Arias, John Buckell, Marian Knight, B M Craig, Rema Ramakrishnan, Simon Kenny, Benjamin Allin

**Affiliations:** 1 Nuffield Department of Population Health, National Perinatal Epidemiology Unit (NPEU), University of Oxford, Oxford, UK; 2 Nuffield Department of Population Health, Health Economics Research Centre (HERC), University of Oxford, Oxford, UK; 3 Department of Economics, University of South Florida, Tampa, Florida, USA; 4 Department of Paediatric Surgery, Alder Hey Children's Hospital, Liverpool, UK; 5 Chelsea and Westminster Hospital, London, UK

**Keywords:** health care economics and organizations, health services research, paediatrics

## Abstract

**Objectives:**

Develop a score summarising how successfully a child with any surgical condition has been treated, and test the clinical validity of the score.

**Design:**

Discrete choice experiment (DCE), and secondary analysis of data from six UK-wide prospective cohort studies.

**Participants:**

253 people with lived experience of childhood surgical conditions, 114 health professionals caring for children with surgical conditions and 753 members of the general population completed the DCE. Data from 1383 children with surgical conditions were used in the secondary analysis.

**Main outcome measures:**

Normalised importance value of attribute (NIVA) for number/type of operations, hospital-treated infections, quality of life and duration of survival (reference attribute).

**Results:**

Quality of life and duration of survival were the most important attributes in deciding whether a child had been successfully treated. Parents, carers and previously treated adults placed equal weight on both attributes (NIVA=0.996; 0.798 to 1.194). Healthcare professionals placed more weight on quality of life (NIVA=1.469; 0.950 to 1.987). The general population placed more weight on survival (NIVA=0.823; 95% CI 0.708 to 0.938). The resulting score (the Children’s Surgery Outcome Reporting (CSOR) Treatment Success Score (TSS)) has the best possible value of 1, a value of 0 describes palliation and values less than 0 describe outcomes worse than palliation. CSOR TSSs varied clinically appropriately for infants whose data were included in the UK-wide cohort studies.

**Conclusions:**

The CSOR TSS summarises how successfully children with surgical conditions have been treated, and can therefore be used to compare hospitals’ observed and expected outcomes.

**WHAT IS ALREADY KNOWN ON THIS TOPIC**

- Unwarranted variation between hospitals in England and Scotland exists in the management and outcomes of children undergoing specialised surgery.
- Improving outcomes for children with surgical conditions requires an understanding of whether hospitals’ observed outcomes differ to their expected outcomes.
- To determine differences between hospitals’ observed and expected outcomes, it is necessary to combine data from children with a broad range of surgical conditions.

**WHAT THIS STUDY ADDS**

- Quality of life and duration of survival are the most important determinants of how successfully a child with a surgical condition has been treated.
- The number and type of operations and the number of times children have been treated in hospital for an infection contribute significantly less to determining treatment success.
- This study has developed a score of the success of treatment (Children’s Surgery Outcome Reporting (CSOR) Treatment Success Score (TSS)).

**HOW THIS STUDY MIGHT AFFECT RESEARCH, PRACTICE OR POLICY**

- The CSOR TSS can now be used to determine whether hospitals’ observed outcomes for children with surgical conditions differ to their expected outcomes.
- This information can be used by hospitals to help understand how their management strategies may be influencing their outcomes.

## Introduction

Approximately half a million children undergo an operation each year in the UK.^1^ The National Confidential Enquiry into Patient Outcomes and Death report into the organisational and clinical aspects of children’s surgery,^1^ the Getting It Right First Time report^2^ and 15 years of the UK-wide BAPS-CASS cohort studies^3–16^ have provided evidence that unwarranted variation between hospitals exists in the management and outcomes of children undergoing specialised surgery.

Reducing unwarranted variation requires understanding whether hospitals’ observed and expected outcomes differ, and by extension, whether management strategies used in individual hospitals are delivering the best possible care for children. The low incidence of childhood surgical conditions complicates the analysis of observed versus expected outcomes when conditions are studied in isolation. Combining data across a range of conditions can enable reliable comparisons. To meaningfully combine data across multiple surgical conditions of childhood, an outcome measure that describes how successfully a child with a surgical condition has been treated is required. Such an outcome measure has not previously been developed.

The overall objective of this study was therefore to understand how to combine, into a single summary measure describing treatment success, key attributes of the outcome of a child with a surgical condition. A secondary objective was to demonstrate the implementation of this summary measure using existing observational data. This study was conducted as part of the National Institute for Health Research (NIHR)-funded Children’s Surgery Outcome Reporting (CSOR) Programme.

## Methods

### Discrete choice experiment study summary

Discrete choice experiments (DCEs) are commonly used to determine participants’ preferences for treatment options and health states.^17^ Within a DCE, different ‘attributes’ (eg, ‘quality of life’) are used to describe ‘scenarios’ (eg, ‘treatment A’ or ‘treatment B’), and participants are asked to trade off between (usually) pairs of these scenarios in a series of ‘tasks’. The participants’ responses to DCE tasks can be used to determine the relative value they put on the attributes described in the scenarios.

To develop a summary measure describing how successful the treatment of a child with a surgical condition has been, a DCE was conducted in which participants responded to 10 tasks. Each scenario used seven attributes to describe a child’s health and well-being following treatment for a surgical condition (table 1 and online supplemental figure 1). Participants were asked to select the scenario in each task that they felt described the more successful treatment. The analysis of participants’ choices gave the ‘value’ (i.e., contribution) of each attribute to the measure of treatment success. These values were used to generate a single score which describes how successful a child’s treatment had been. The DCE is summarised below, with extended details in the published protocol.^18^

10.1136/archdischild-2023-326156.supp1 Supplementary data

**Table 1:** Study descriptive framework with final list of attributes and attribute levels

Attributes	Attribute levels	Icon description
Planned major operations related to the condition	No planned major operations	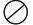
	One planned major operation	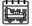
	Two planned major operations	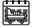 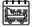
	Six planned major operations	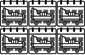
Planned minor operations related to the condition	No planned minor operations	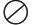
	One planned minor operation	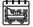
	Two planned minor operations	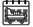 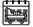
	Six planned minor operations	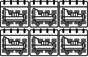
Emergency major operations related to the condition	No emergency major operations	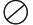
	One emergency major operation	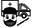
	Two emergency major operations	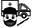 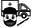
	Six emergency major operations	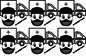
Emergency minor operations related to the condition	No emergency minor operations	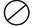
	One emergency minor operation	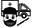
	Two emergency minor operations	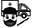 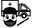
	Six emergency minor operations	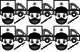
Infections treated in hospital	No infections treated in hospital	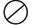
	One infection treated in hospital	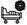
	Two infections treated in hospital	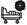 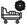
	Six infections treated in hospital	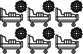
Child’s quality of life	Good quality of life	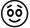
	Fair quality of life	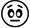
	Poor quality of life	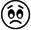
How long the child survived after their diagnosis	More than 20 years, without any expectation that their surgical condition would shorten their life expectancy	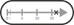
	20 years	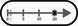
	5 years	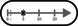
	1 year	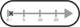
	6 months	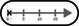
	1 month	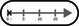

### Identification of the attributes to describe treatment success (the descriptive system)

Attributes and levels were identified by:

1. Reviewing core outcome sets relevant to paediatric surgery.
2. Semistructured group discussions.
3. Think-aloud exercises with parents of children with a surgical condition.

Further details are provided in the online supplemental materials.

### Experimental design

The experimental design (the process of selecting the pairs of scenarios (tasks) to present participants in the DCE) was D-efficient and included 45 choice tasks (online supplemental figure 2).^19^ Participants completed nine choice tasks and an additional palliative choice task. The latter included one scenario describing the outcome of a child’s treatment, and one describing a child being palliated. In the ‘palliative’ scenario, the child underwent no operations, had no infections, had a fair quality of life and survived for 1 month. Further details are provided in the online supplemental materials.

### Survey instrument and recruitment

The survey started with a welcome, three screening questions, the study aims and descriptions of the DCE’s attributes. Participants could watch short videos explaining the study and attributes as an alternative to reading the text descriptions. To aid familiarisation, participants completed short warm-up tasks and a practice choice task prior to completing the 10 study choice tasks. Only participants 18 years and over were eligible to participate.

Three groups of stakeholders were sampled:

1. Parents of children with a surgical condition and people who had been treated for a surgical condition as a child.
2. Healthcare professionals caring for children with surgical conditions.
3. Members of the general population.

A pilot study with stakeholders from groups 1 and 2 was conducted in August 2021, with main data collection between October 2021 and April 2022.

## Data analysis

### Discrete choice modelling

Responses to the choice tasks were summarised using choice probabilities. Experimental choices were modelled using mixed multinomial logit models (see technical details in the online supplemental material). All attributes were included using dummies in the model specification with constraints for inconsistent attribute levels. An additional term was introduced in the model to capture the value of the palliative scenario that was used to estimate the model for the final algorithm.

### Normalised importance value of attributes

Normalised importance value of attributes (NIVAs) express the importance that participants place on one attribute relative to another prespecified attribute. NIVAs were estimated using survival as the reference.^20^ An NIVA of 1 suggests that two attributes are equally important, while NIVAs less than 1 suggest that the comparison attribute is less important than survival and NIVAs greater than 1 suggest the comparison attribute is more important than survival. CIs for NIVAs were estimated using the delta method with pvalues of <0.05 considered statistically significant. NIVAs were calculated separately for each stakeholder group.

### Development of the CSOR Treatment Success Score

A multistakeholder online focus group was convened to determine which stakeholder group preferences to use in the CSOR Treatment Success Score (TSS). The final selected stakeholder preferences were used to estimate a set of coefficients placed on a ‘palliative space scale’. Placement on the palliative space scale creates a score in which a value of 1 represents the best possible set of outcomes (no operations, no infections, good quality of life and survival more than 20 years), a score of 0 represents a scenario considered a close approximation of palliation (no operations, no infection, fair quality of life and survival less than a month) and scores below 0 represent outcomes considered to be worse than palliation. Further details are provided in the online supplemental materials.

### Application of the CSOR TSS to existing data

Data from six UK-wide cohort studies describing the management and outcomes of children with Hirschsprung’s disease, gastroschisis, necrotising enterocolitis (NEC), posterior urethral valve (PUV), congenital diaphragmatic hernia and oesophageal atresia were combined into a single dataset. In the original cohorts, data were collected at birth, 28 days of age and 1 year of age for all conditions. For children with Hirschsprung’s disease and gastroschisis, quality of life data were also collected at primary school age (5–8 years of age). To calculate the CSOR TSS at 1 year of age, quality of life was simulated for all infants through random allocation to good, fair or poor quality of life in the same proportions as was seen in the subset of infants with QoL data at 5–8 years of age. These data were used to calculate:

1. CSOR TSS (including simulated quality of life) at 1 year of age.
2. Partial CSOR TSS (excluding quality of life) at 1 year of age.
3. CSOR TSS at 5–8 years of age using data only for those infants in whom quality of life data were available.

All calculated CSOR TSSs were described according to key infant characteristics, including primary diagnosis, gestational age at birth, birth weight, ethnicity, sex, presence of an additional major congenital anomaly and maternal age.

### Patient and public involvement

The CSOR Programme has established a Parent Advisory Group (PAG) consisting of over 100 parents and families of children who have undergone early surgery for conditions including Hirschsprung’s disease, gastroschisis, exomphalos, short bowel and NEC. The PAG was pivotal during the study design and the interpretation of results of the DCE. They were actively involved from the point of study conception, through identification of attributes and levels, design of the study instrument including refinement of language and presentation, and through to interpretation and explanation of the results.

## Results

### Respondents

Table 2 and online supplemental table 1 describe the characteristics of the 1120 people completing the DCE.

**Table 2:** Characteristics of the study participants included in each stakeholder group

	Parent or carer or treated as a child	Healthcare professional	General population
	n=253	n=114	n=753
Age, mean (SD)	45.3 (14.9)	42.3 (9.9)	50.1 (17.0)
Gender			
Female	192 (75.9%)	66 (57.9%)	412 (54.7%)
Male	61 (24.1%)	47 (41.23%)	332 (44.1%)
Other	0 (0.0%)	0 (0.0%)	4 (0.5%)
Prefer not to say	0 (0%)	1 (0.88%)	5 (0.7%)
Region			
England: North East			33 (4.4%)
England: North West			80 (10.6%)
England: Yorkshire and the Humber			59 (7.8%)
England: East Midlands			56 (7.4%)
England: West Midlands			65 (8.6%)
England: East of England			72 (9.6%)
England: London			92 (12.2%)
England: South East			114 (15.1%)
England: South West			65 (8.6%)
Northern Ireland			19 (2.5%)
Scotland			57 (7.6%)
Wales			41 (5.4%)
Ethnicity			
Asian and Asian British			56 (7.4%)
Black, black British, Caribbean or African			16 (2.1%)
Mixed or multiple ethnic groups			4 (0.5%)
Prefer not to answer			9 (1.2%)
White (British, Irish, Gypsy or Irish Traveller, Roma)			625 (83.0%)
White (other)			43 (5.7%)
Higher education			
Yes	158 (62.5%)		
No	95 (37.5%)		
Highest education			
Postgraduate degree or above/NVQ level 7–8			104 (13.8%)
Undergraduate degree/diploma/NVQ level 5–6			230 (30.5%)
A levels or Scottish Highers/HNC or equivalent/NVQ level 3–4			382 (50.7%)
None of the above			29 (3.9%)
Prefer not to answer			8 (1.1%)
Healthcare professional			
Yes			29 (3.9%)
No			724 (96.1%)
Are you a parent or carer?			
Yes			398 (52.9%)
No			355 (47.1%)
Occupational group main earner in household			
Administrative and secretarial occupations	27 (10.7%)		112 (14.9%)
Caring, leisure and other service occupations	14 (%)		24 (3.2%)
Elementary occupations	3 (1.1%)		17 (2.3%)
Managers, directors and senior officials	49 (19.4%)		99 (13.1%)
Never employed	0 (0.0%)		5 (0.7%)
Pensioner	26 (10.3%)		145 (19.3%)
Process, plant and machine operatives	3 (1.1%)		13 (1.7%)
Professional/associate professional occupations	70 (27.7%)		135 (17.9%)
Sales and customer service occupations	8 (3.2%)		47 (6.2%)
Skilled trades occupations	30 (11.9%)		72 (9.6%)
Unemployed	14 (5.5%)		53 (7.0%)
Prefer not to answer	9 (3.6%)		31 (4.1%)
Marital status			
Divorced	5 (1.9%)		69 (9.2%)
Married/partner	126 (49.8%)		426 (56.6%)
Separated	3 (1.2%)		8 (1.1%)
Single	9 (3.6%)		215 (28.6%)
Widowed	4 (1.6%)		29 (3.9%)
Missing	106 (41.9%)		0 (0.0%)
Prefer not to answer	0 (0.0%)		6 (0.8%)
Have you experienced serious illness?*†			
Yes	68 (26.9%)	18 (15.8%)	176 (23.4%)
No	177 (70.0%)	92 (80.7%)	562 (74.6%)
Prefer not to answer	8 (3.2%)	4 (3.5%)	15 (2.0%)
Have you experienced serious illness in your family?*†			
Yes	126 (49.8%)	63 (55.3%)	394 (52.3%)
No	121 (47.8%)	47 (41.2%)	338 (44.9%)
Prefer not to answer	6 (2.4%)	4 (3.5%)	21 (2.8%)
Have you experienced serious illness as a carer for a family member?*†			
Yes	64 (25.3%)	27 (23.7%)	130 (17.3%)
No	185 (73.1%)	83 (72.8%)	602 (79.9%)
Prefer not to answer	4 (1.6%)	4 (3.5%)	21 (2.8%)
What is your role/specialism?			
Surgeon		36 (31.6%)	
Anaesthetist		34 (29.8%)	
Paediatrician		7 (6.1%)	
Neonatologist		10 (8.8%)	
Nurse		12 (10.5%)	
Advanced nurse practitioner/specialist nurse		10 (8.8%)	
Other		1 (0.9%)	
Missing		4 (3.5%)	
Highest current level of training (for surgeon, anaesthetist, paediatrician and neonatologist)			
Consultant or equivalent		66 (57.9%)	
Registrar or equivalent		19 (16.7%)	
Foundation or core trainee		2 (1.8%)	
Missing		27 (23.7%)	
Age category of child†			
My child has died	4 (2.7%)		
Less than 1 year old	6 (4.1%)		
1–5 years old	46 (31.3%)		
6–10 years old	35 (23.8%)		
11–18 years old	35 (23.8%)		
More than 18 years old	21 (14.3%)		
Proxy-report of your child’s quality of life†			
Good quality of life	113 (76.9%)		
Fair quality of life	28 (19.1%)		
Poor quality of life	1 (0.7%)		
Prefer not to answer	1 (0.7%)		
Missing	4 (2.7%)		

Data are n (%) unless otherwise specified. Empty cells indicate question was not included in the demographics for that stakeholder.

*These questions included ‘in additional to having a child with a surgical condition’ in the survey administered to parents and carers, and people treated for surgical conditions as a child.

†Information only collected for parents or carers of a child (n=147).

HNC, Higher National Certificate; NVQ, National Vocational Qualification.

### Stakeholder preferences

**Figure 1:**
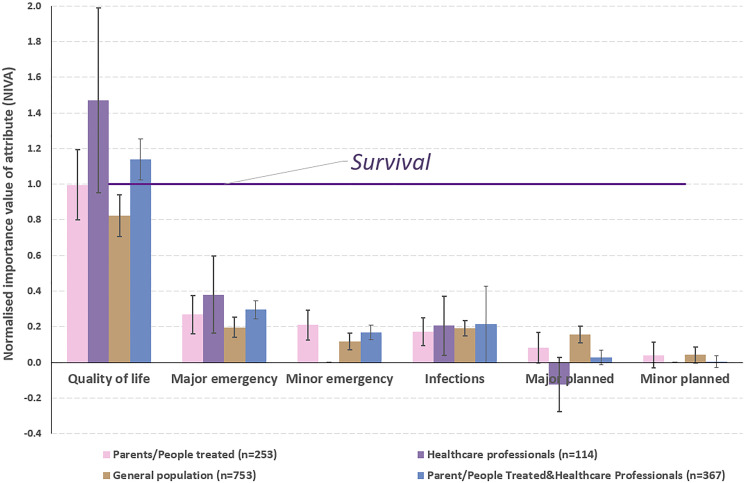
Normalised importance value of attribute with associated 95% CIs presented for each stakeholder group separately and combined for parent/people treated and healthcare professionals. Survival attribute is the reference.

Choice probabilities for each choice task provided a preliminary indication that quality of life and survival were the most important attributes in deciding whether a child had been successfully treated (online supplemental table 2). This was corroborated across all stakeholder groups in the modelling exercise (figure 1 and online supplemental table 3). Parents, carers and previously treated adults placed equal weight on quality of life and survival (NIVA=0.996; 0.798 to 1.194). Healthcare professionals placed more weight on quality of life than survival (NIVA=1.469; 0.950 to 1.987) (figure 1). The general population placed less weight on quality of life than survival (NIVA=0.823; 95% CI 0.708 to 0.938). Emergency operations and infections had some bearing on how successfully a child was considered to have been treated, but this influence was considerably less than that of quality of life and survival. Planned operations had a negligible impact on how successful a child’s treatment was considered to be. These results were similar across all stakeholder groups (figure 1). See online supplemental table 3 for the coefficients of the mixed multinomial logit model from which the NIVAs were derived.

### Combining preferences across stakeholder groups

The focus group felt that there were clear differences in the DCE-estimated preferences between the stakeholder groups, particularly around the value placed on quality of life. They agreed that as the CSOR TSS will be used to compare hospitals’ observed with expected outcomes, the preferences of those with direct experience of healthcare in these settings should be used for development of the CSOR TSS over and above the preferences of those who could potentially experience healthcare in these settings in the future. The preferences of the general population were therefore not used in development of the final CSOR TSS algorithm.

### CSOR Treatment Success Score

**Figure 2:**
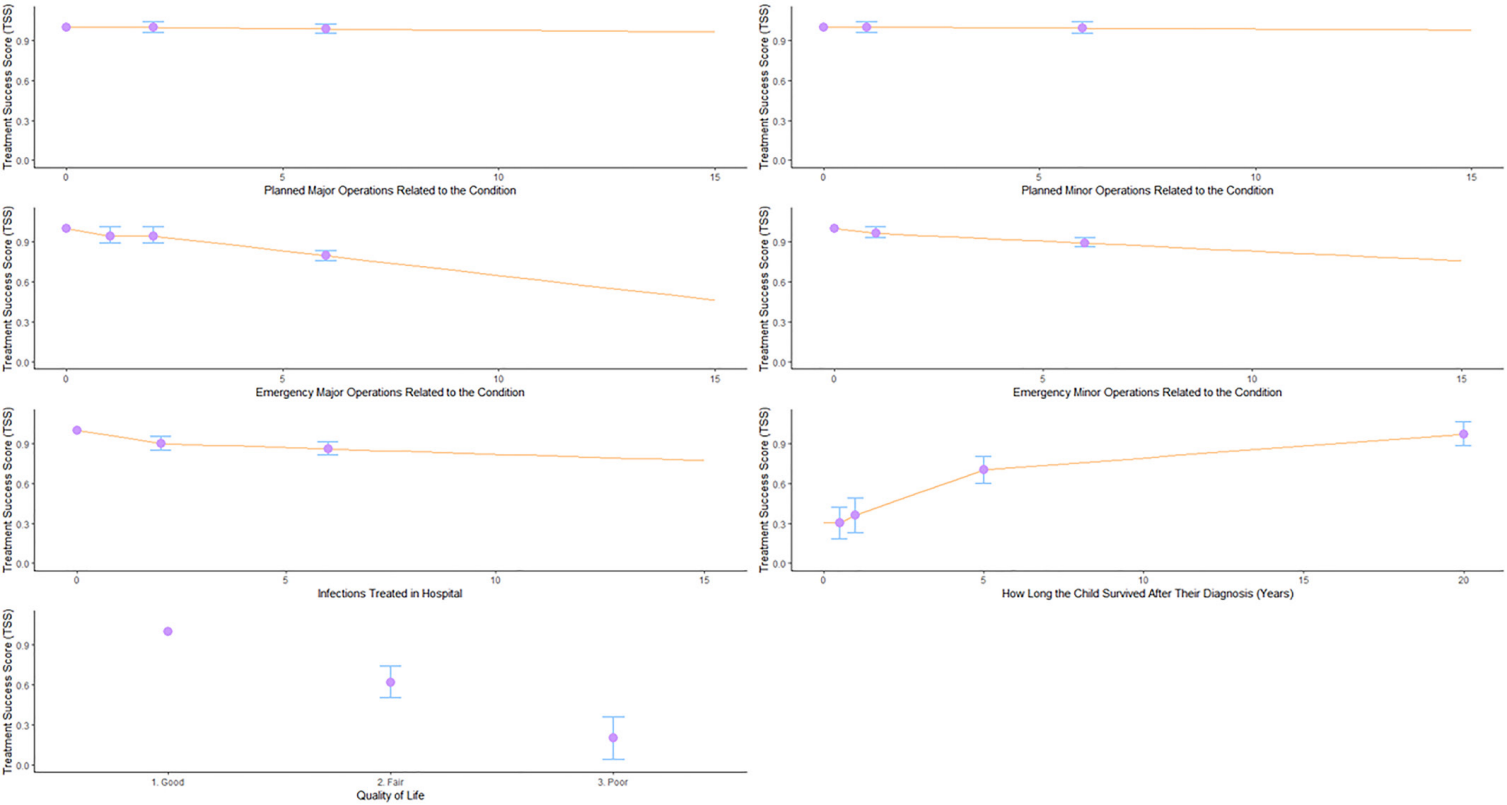
Children’s Surgery Outcome Reporting Treatment Success Score and impact of number/type of operations, hospital-treated infections, quality of life and survival.

Based on the Palliative Space Scale model (online supplemental table 3), reduced quality of life and duration of survival were associated with large utility decrements, and therefore both of these attributes significantly impact the CSOR TSS. Figure 2 describes the utility decrements for each attribute. The overall CSOR TSS for a given scenario is calculated by summing the utility decrements associated with the attribute levels described in that scenario and subtracting them from 1. See online supplemental figure 3 for examples.

### Application of the CSOR TSS

The mean CSOR TSS (including simulated quality of life) at 1 year of age varied across conditions, ranging from 0.53 (SD 0.44) in children with NEC, to 0.75 (SD 0.27) in children with PUV. The CSOR TSS also varied in a clinically expected way across gestational age at birth, birth weight, maternal age, ethnicity, and according to the presence or absence of additional congenital anomalies (table 3). When CSOR TSSs (including real quality of life data) were described at 5–8 years of age for children with Hirschsprung’s disease and gastroschisis, and when partial CSOR TSSs (excluding quality of life) were described at 1 year of age for all children in the cohort, similar patterns of clinically expected variation in scores across characteristics were observed (online supplemental tables 4 and 5).

**Table 3:** Mean CSOR TSS at 1 year of age by characteristics of infants (calculated using simulated quality of life with population with same proportion as BAPS-CASS data (11% poor, 38% fair, 51% good))

Variables	n (%)	Mean (SD)
Overall score	1383	0.66 (0.36)
Surgical condition		
CDH	212 (15)	0.59 (0.44)
Gastroschisis	353 (26)	0.71 (0.30)
HD	305 (22)	0.73 (0.29)
NEC	243 (18)	0.53 (0.44)
OA	151 (11)	0.69 (0.36)
PUV	119 (9)	0.75 (0.27)
Associated anomalies		
No	1054 (77)	0.67 (0.35)
Yes	319 (23)	0.64 (0.39)
Missing	10	
Sex		
Female	716 (52)	0.68 (0.34)
Male	663 (48)	0.65 (0.38)
Missing	4	
Birth weight, g		
≥2500	754 (56)	0.70 (0.33)
<2500	585 (44)	0.61 (0.39)
Missing	44	
Gestational age, weeks		
<28	143 (10)	0.48 (0.47)
28–<32	91 (7)	0.57 (0.42)
32–<37	305 (22)	0.66 (0.36)
≥37	824 (60)	0.71 (0.32)
Missing	20	
Ethnicity		
White	1121 (84)	0.68 (0.35)
Black	58 (4)	0.50 (0.46)
Asian	103 (8)	0.67 (0.37)
Mixed/other	58 (4)	0.64 (0.34)
Missing	43	
Maternal age, years		
<20	151 (12)	0.72 (0.31)
20–<25	288 (24)	0.65 (0.35)
25–<30	283 (23)	0.66 (0.36)
30–<35	275 (22)	0.67 (0.37)
≥35	221 (18)	0.61 (0.42)
Missing	165	

140 of 143 infants who were less than 28 weeks of gestation at birth had NEC.

CDH, congenital diaphragmatic hernia; CSOR, Children’s Surgery Outcome Reporting; HD, Hirschsprung’s disease; NEC, necrotising enterocolitis; OA, oesophageal atresia; PUV, posterior urethral valve; TSS, Treatment Success Score.

## Discussion

### Summary of key findings

This study developed a summary measure describing how successfully a child with a surgical condition has been treated (the CSOR TSS). The CSOR TSS is based on the child’s duration of survival and quality of life, which were the most important determinants of treatment success, as well as the number and type of operations the child has undergone, and the number of times they have been treated in hospitals for an infection related to their surgical condition.

### Strengths and weaknesses

The key strength of this study is its substantial use of personal, parent and healthcare professional experiences channelled through well-established elicitation techniques and statistical modelling. Over 11,000 pairwise comparisons were conducted in the DCE to understand the relative importance of the attributes to different stakeholder groups. The DCE results were reviewed with parents and healthcare professionals to ensure that there was a coherent clinical logic to them and that they reflected real-world experiences. We combined the DCE results with data from six separate observational studies providing CSOR TSSs across multiple surgical conditions. The subsequent testing of the CSOR TSS demonstrated that it appropriately discriminates children whose outcomes are expected to be worse.

Only 114 healthcare professionals completed the DCE survey, and it is therefore possible that the results do not fully reflect the views of healthcare professionals. Due to the dominance of quality of life and survival over the other attributes, the experimental design did not allow estimation of coefficients for some levels of the operation and hospital-treated infection attributes. It is clear from the results, however, that these attributes were only minor drivers of treatment success and this limitation is therefore unlikely to have biased the results.

The CSOR TSS is intended to be used to describe treatment success for ‘a child with a surgical condition’. Consequently, the descriptive system focused on outcomes that are important across a range of different styles of surgical condition and we believe that the resulting measure of treatment success is also likely to be relevant across a broader range of surgical conditions. Despite this, the validation of the TSS has only taken place on a small subset of neonatal surgical conditions, as these are the low case number, high-complexity conditions where the use of the TSS will be most beneficial, and for which the most reliable data are currently available. Further studies will be required to validate if the TSS is also applicable to other areas of paediatric surgery, particularly paediatric oncology where there is a combined medical-surgical approach, and in which the contribution of surgery to outcome may be harder to discern. In addition, this approach is less likely to be as beneficial in low-complexity high-volume paediatric surgical practice where it may be feasible to use other quantitative analyses to detect unwarranted variation between hospitals in management and outcome.

### Comparison with previous literature

We are not aware of any other example in which a DCE has been used to develop an algorithm for determining how successfully people have been treated. Success of surgical treatment is more commonly determined by crude metrics including readmission to hospital, length of stay and operative time.^21 22^ Condition-specific outcomes such as faecal incontinence for children with Hirschsprung’s disease or stricture formation for children with oesophageal atresia have also previously been used as determinants of success.^21 23–27^ However, such metrics and specific outcomes are not ideal for determining how successfully an individual with *any* surgical condition has been treated. Many of the crude metrics have been shown repeatedly to be unimportant in determining treatment success^28–33^ while the condition-specific outcomes will only ever be relevant to a subset of infants, and when used on their own may give an incomplete picture of the outcome of the child’s treatment.

### Implications and conclusions

The NIHR-funded CSOR Programme is running over 5 years with an aim of understanding whether it is possible to identify and reduce unwarranted variation in management and outcome of children treated for surgical conditions in 10 pilot hospitals across England and Scotland. Development of the CSOR TSS has made it possible to meaningfully combine outcomes data for children with a range of surgical conditions, and therefore determine whether outcomes in these 10 pilot hospitals are better or worse than would be expected based on the case-mix of children they have treated. This information is essential for identifying and reducing unwarranted variation in management and outcome.

## Data Availability

Data are available upon reasonable request.
