# Supplementary material for: Defining treatment success in children with surgical conditions
Source: Arch Dis Child. 2023 Dec 22;109(5):377–86. doi: 10.1136/archdischild-2023-326156 (PMC11041596; doi:10.1136/archdischild-2023-326156)
Supplement: Supplementary data [file archdischild-2023-326156supp001.pdf]

Supplementary material for "Defining treatment success for children with surgical conditions using preferences from key stakeholders: a discrete choice experiment study"

Table of contents

Supplementary material identification of the attributes to describe treatment success (the descriptive system) 2
Supplementary material experimental design 3
Supplementary material discrete choice modelling methods 4
Supplementary material development of the CSOR treatment success score (TSS) 5
Figure S1: Example of choice task completed by participants 6
Figure S2: Choice tasks identified in the experimental design (tasks 1-45) and choice tasks including palliative scenario (choice tasks 46-60) 7
Figure S3: Examples of CSOR Treatment Success Score (TSS) 16
Table S1: Treated conditions of children and participants treated as a child completing the DCE exercise 17
Table S2: Mean choice probabilities associated to each choice task by stakeholder group. Values indicate the probability of selecting scenario B 18
Table S3: Mixed multinomial logit model estimates of choice responses for stakeholders. 20
Table S4: Mean scores by characteristics of infants with HD and gastroschisis who had QoL data at 5-8 years of age 22
Table S5: Mean scores by characteristics of infants across the fulls sample with partial CSOR TSSs (excluding quality of life) 23
References: 24

Rivero-Arias O, *et al. Arch Dis Child* 2024; 109:377–386. doi: 10.1136/archdischild-2023-326156

Supplementary material identification of the attributes to describe treatment success (the descriptive system)

The literature was reviewed to identify published core outcome sets (COS) relevant to paediatric surgery. COS for Hirschsprung's disease, gastroschisis, appendicitis, and neonatal care in high-income countries were identified.[1-4] The outcomes contained within the four COS were reviewed by:

- The study team
- A surgical advisory group consisting of consultant paediatric surgeons representing eight of the 24 hospitals providing specialised surgery for children in England and Scotland, and
- A parental advisory group consisting of parents of children who have undergone early surgery, as well as charity and support group representatives.

Review of outcomes by the parental and surgical advisory groups took the form of online, semi-structured group discussions, chaired by MK. This format of online focus groups was utilised throughout the study when stakeholder input was required to aid study design or interpretation of results. The outcome review focus groups concluded that there were outcomes common across the COSs which could be represented by four summary attributes. These were, the length of time the child survived for, the number and type of operations the child underwent, the number of times they were treated in hospital for an infection related to their surgical condition, and their overall quality of life. This work also identified that although there were condition specific outcomes, such as faecal incontinence, reported in the COSs, these were outcomes that were highly likely to impact the quality of life of a child, and therefore, within the descriptive system for the DCE, they could be adequately represented by the attribute 'quality of life'. To ensure the clinical plausibility of the 'levels' that were assigned to each attribute in the DCE scenarios, the existing literature was reviewed to identify plausible durations of survival, numbers and types of operations, numbers of infections, and quality of life, for six key conditions (Hirschsprung's disease, gastroschisis, congenital diaphragmatic hernia (CDH), necrotising enterocolitis (NEC), posterior urethral valves (PUV) and oesophageal atresia) falling under the remit of specialised surgery in children. The combination of attributes and levels that are used within a DCE is referred to as the descriptive system. To test the clinical plausibility and understandability of the developed descriptive system, this was reviewed in a further online session with the surgical advisory group. Three think-aloud exercises were also conducted with parents who completed a mock version of the DCE with two of the researchers (OR-A, JB). Both the surgical advisory group and the parents were given the opportunity during these sessions to comment on the wording used for the attributes and associated levels, the presentation of the descriptive system, and the usability of the DCE interface. Further details of the identification and definition of attributes and attribute levels for this DCE are described in the original protocol.[5]

Supplementary material experimental design

An initial candidate list of 45 choice tasks was generated using Ngene software (Figure S2).[6] This candidate list was divided into five blocks of nine choice tasks, with each participant randomised to one of these blocks. The order of appearance of the choice tasks within blocks was randomly generated for each participant.

To facilitate completion of the choice tasks and to reduce participant fatigue, no implausible scenarios were included in the candidate list, and within each choice task, the levels for a minimum of three attributes were the same in both scenarios. The identical attributes were shaded in grey (Figure S1). Graphics as well as words were used to describe the attributes as this has previously been shown to improve choice consistency (Table 1 main text).[7] The final candidate set was revised by the research team (OR-A, JB, BA) to ensure that no single scenario was dominant or dominated in each of the pairs.

Supplementary material discrete choice modelling methods

Our experimental design and candidate set assumed that all attributes in the DCE were important and contributed to the overall value of treatment success of a child with a surgical condition. However, it was expected a priori that the attributes quality of life and survival would make the largest contribution of the value of treatment success. As we did not have any evidence of the magnitude of how more important these two attributes were with respect to operations and hospital-related infections, no adjustment for this was introduced in the experimental design. Our pilot DCE study confirmed our hypothesis that survival and quality of life were going to be the most important attributes with significant coefficients in the model for these two attributes but insignificant coefficients for the remaining attributes using the limited sample size in the pilot. However, one artefact of our experimental design that was not detected during the piloting phase was that when a scenario described a profile of better quality of life and survival than the alternative scenario, the scenario with a better profile tended to have significantly more one major planned operation, two minor planned operations, two minor emergency operations and one hospital-treated infection than the alternative profile. This resulted in positive coefficients in our discrete choice models and evidence of logical inconsistency in the expected direction in those compromised attribute levels. We were certain that this could not represent a real preference because of 1) the remaining attribute levels for the attribute were logically consistent with the expected sign and 2) the same behaviour was observed in each of the three sets of stakeholder preferences collected in the study. Unfortunately, and primarily because the coefficients associated to the compromised attribute levels were not statistically significant during the pilot study, the research team only realised about this after data collection has been completed in the main study. This was resolved by collapsing some of the compromised attribute levels to the previous closest level in that attribute or zero.

Models were estimated in Stata using 5,000 Halton draws and the Broyden-Fletcher-Goldfarb-Shanno (BFGS) algorithm and in R using the Apollo package and 5,000 Modified Latin Hypercube Sampling draws and the BFGS algorithm.

Supplementary material development of the CSOR treatment success score (TSS)

A multi-stakeholder focus group consisting of eight paediatric surgeons, three parents/carers of children with surgical conditions, three social anthropologists, one statistician and one health economist was chaired by MK. Within this meeting, the results of the DCE were reviewed to determine the most appropriate combination of stakeholder preferences from which to derive the final CSOR Treatment Success Score (TSS). The final selected stakeholder preferences were used to estimate a set of coefficients placed on a “palliative space scale”; that is, all coefficients expressed relative to the value of the palliative scenario. The model was a multinomial mixed logit in which the coefficients are all divided by the coefficient of the palliative scenario. This was operationalised in R using the willingness-to-pay space specification of Train and Weeks.[8] Using this approach, the attribute level coefficients of this model are placed on a scale anchored at two points, such that a score of 1 represented the best possible scenario (no operations, no infections, good quality of life, and survival more than 20 years), and a score of 0 represented a scenario considered a close approximation of palliation (no operations, no infection, fair quality of life, and survival less than a month). The derived model was used to calculate ‘utility decrements’ for each attribute in the DCE. A utility decrement is a numeric value given to a specific level of an attribute e.g. survival for 1 year, that describes how much worse this level is considered to be than the best possible level of that attribute e.g. survival more than 20 years. The larger the utility decrement, the worse the level is considered to be. A series of linear and nonlinear models were used to capture the relationship between successful outcomes of an operation (i.e. the choice in the choice tasks) and the number of operations, hospital-based infections, and length of survival. The fits of these models were considerably worse than their dummy-coded equivalents and the latter were retained. To apply the algorithm with a dummy-coded model, linear interpolation was applied, with Euclidean geometry between attribute levels determining the gradient. In some cases where the parameter estimates were very similar in magnitude (not distinguishable with statistical testing), a higher parameter for a higher attribute level was estimated (e.g. for 3 versus 2 major operations). The implication for the algorithm is that that TSS increases moving from level 2 to level 3, which was an undesirable feature of the algorithm. To correct for these cases, the lower estimate was used in the algorithm for both levels.

Figure S1: Example of choice task completed by participants

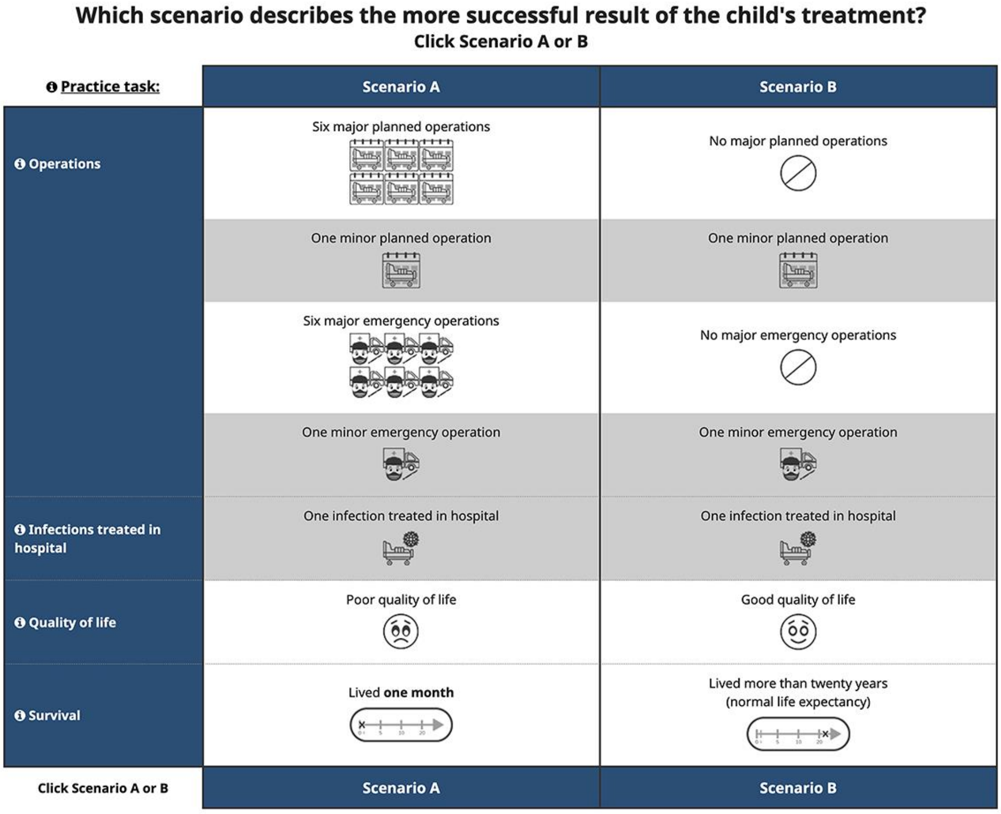

Figure S2: Choice tasks identified in the experimental design (tasks 1-45) and choice tasks including palliative scenario (choice tasks 46-60)

Scenario 1

	Scenario A	Scenario B
**Planned major**	Two planned major	Six planned major
**Planned minor**	Six planned minor	Six planned minor
**Emergency major**	Two emergency major	Two emergency major
**Emergency minor**	Six emergency minor	No emergency minor
**Infections**	Six infections	Two infections
**Quality of life**	Poor	Poor
**Survival**	1 year	6 months

Scenario 2

	Scenario A	Scenario B
**Planned major**	No planned major	One planned major
**Planned minor**	Two planned minor	No planned minor
**Emergency major**	Six emergency major	No emergency major
**Emergency minor**	One emergency minor	One emergency minor
**Infections**	Six infections	Six infections
**Quality of life**	Fair	Poor
**Survival**	More than 20 years	More than 20 years

Scenario 3

	Scenario A	Scenario B
**Planned major**	No planned major	Six planned major
**Planned minor**	No planned minor	No planned minor
**Emergency major**	No emergency major	Six emergency major
**Emergency minor**	Two emergency minor	One emergency minor
**Infections**	Six infections	Two infections
**Quality of life**	Good	Good
**Survival**	More than 20 years	More than 20 years

Scenario 4

	Scenario A	Scenario B
**Planned major**	One planned major	Six planned major
**Planned minor**	No planned minor	No planned minor
**Emergency major**	Two emergency major	Six emergency major
**Emergency minor**	Two emergency minor	Two emergency minor
**Infections**	Six infections	Two infections
**Quality of life**	Poor	Poor
**Survival**	6 months	More than 20 years

Scenario 5

	Scenario A	Scenario B
**Planned major**	Six planned major	Six planned major
**Planned minor**	One planned minor	Two planned minor
**Emergency major**	Six emergency major	Six emergency major
**Emergency minor**	Two emergency minor	No emergency minor
**Infections**	Six infections	Six infections
**Quality of life**	Good	Poor
**Survival**	1 year	5 years

Scenario 6

	Scenario A	Scenario B
**Planned major**	Six planned major	Six planned major
**Planned minor**	No planned minor	No planned minor
**Emergency major**	One emergency major	Six emergency major
**Emergency minor**	Six emergency minor	One emergency minor
**Infections**	Two infections	Six infections
**Quality of life**	Poor	Poor
**Survival**	5 years	20 years

Scenario 7

	Scenario A	Scenario B
**Planned major**	One planned major	One planned major
**Planned minor**	No planned minor	No planned minor
**Emergency major**	One emergency major	Six emergency major

Scenario 8

	Scenario A	Scenario B
**Planned major**	No planned major	Two planned major
**Planned minor**	No planned minor	No planned minor
**Emergency major**	Two emergency major	Six emergency major

**Emergency minor**	Six emergency minor	Two emergency minor
**Infections**	Six infections	Two infections
**Quality of life**	Poor	Poor
**Survival**	More than 20 years	6 months

**Emergency minor**	Two emergency minor	No emergency minor
**Infections**	Six infections	Six infections
**Quality of life**	Poor	Good
**Survival**	More than 20 years	More than 20 years

Scenario 9

	**Scenario A**	**Scenario B**
**Planned major**	Six planned major	Six planned major
**Planned minor**	One planned minor	Two planned minor
**Emergency major**	Two emergency major	One emergency major
**Emergency minor**	No emergency minor	Six emergency minor
**Infections**	Six infections	Six infections
**Quality of life**	Poor	Fair
**Survival**	6 months	6 months

Scenario 10

	**Scenario A**	**Scenario B**
**Planned major**	Six planned major	Six planned major
**Planned minor**	No planned minor	Two planned minor
**Emergency major**	Two emergency major	One emergency major
**Emergency minor**	Six emergency minor	One emergency minor
**Infections**	No infections	No infections
**Quality of life**	Good	Poor
**Survival**	20 years	20 years

Scenario 11

	**Scenario A**	**Scenario B**
**Planned major**	One planned major	One planned major
**Planned minor**	Six planned minor	Six planned minor
**Emergency major**	Two emergency major	Two emergency major
**Emergency minor**	One emergency minor	Six emergency minor
**Infections**	One infection	No infections
**Quality of life**	Poor	Fair
**Survival**	5 years	6 months

Scenario 12

	**Scenario A**	**Scenario B**
**Planned major**	Two planned major	Six planned major
**Planned minor**	Two planned minor	No planned minor
**Emergency major**	No emergency major	One emergency major
**Emergency minor**	Six emergency minor	One emergency minor
**Infections**	Two infections	Two infections
**Quality of life**	Good	Good
**Survival**	More than 20 years	More than 20 years

Scenario 13

	**Scenario A**	**Scenario B**
**Planned major**	Two planned major	Two planned major
**Planned minor**	Two planned minor	Six planned minor
**Emergency major**	Six emergency major	Six emergency major
**Emergency minor**	No emergency minor	Six emergency minor
**Infections**	One infection	One infection
**Quality of life**	Poor	Fair
**Survival**	20 years	More than 20 years

Scenario 14

	**Scenario A**	**Scenario B**
**Planned major**	One planned major	Six planned major
**Planned minor**	One planned minor	Two planned minor
**Emergency major**	Six emergency major	One emergency major
**Emergency minor**	One emergency minor	One emergency minor
**Infections**	Six infections	Six infections
**Quality of life**	Fair	Fair
**Survival**	5 years	More than 20 years

Scenario 15

Scenario 16

	Scenario A	Scenario B
**Planned major**	One planned major	One planned major
**Planned minor**	No planned minor	No planned minor
**Emergency major**	Six emergency major	Six emergency major
**Emergency minor**	No emergency minor	Two emergency minor
**Infections**	One infection	No infections
**Quality of life**	Fair	Poor
**Survival**	6 months	5 years

	Scenario A	Scenario B
**Planned major**	Two planned major	Six planned major
**Planned minor**	Six planned minor	No planned minor
**Emergency major**	Six emergency major	Two emergency major
**Emergency minor**	Six emergency minor	Six emergency minor
**Infections**	One infection	One infection
**Quality of life**	Poor	Poor
**Survival**	More than 20 years	20 years

Scenario 17

	Scenario A	Scenario B
**Planned major**	No planned major	One planned major
**Planned minor**	Six planned minor	One planned minor
**Emergency major**	No emergency major	One emergency major
**Emergency minor**	No emergency minor	No emergency minor
**Infections**	Two infections	No infections
**Quality of life**	Good	Good
**Survival**	More than 20 years	More than 20 years

Scenario 18

	Scenario A	Scenario B
**Planned major**	One planned major	Two planned major
**Planned minor**	Six planned minor	No planned minor
**Emergency major**	One emergency major	One emergency major
**Emergency minor**	One emergency minor	Two emergency minor
**Infections**	No infections	No infections
**Quality of life**	Poor	Poor
**Survival**	1 year	More than 20 years

Scenario 19

	Scenario A	Scenario B
**Planned major**	One planned major	One planned major
**Planned minor**	Two planned minor	Six planned minor
**Emergency major**	Two emergency major	One emergency major
**Emergency minor**	One emergency minor	No emergency minor
**Infections**	No infections	Six infections
**Quality of life**	Poor	Poor
**Survival**	20 years	20 years

Scenario 20

	Scenario A	Scenario B
**Planned major**	One planned major	Six planned major
**Planned minor**	Two planned minor	Two planned minor
**Emergency major**	One emergency major	One emergency major
**Emergency minor**	Two emergency minor	No emergency minor
**Infections**	One infection	No infections
**Quality of life**	Good	Good
**Survival**	More than 20 years	1 year

Scenario 21

	Scenario A	Scenario B
**Planned major**	One planned major	Six planned major
**Planned minor**	Two planned minor	No planned minor
**Emergency major**	Six emergency major	Six emergency major
**Emergency minor**	Six emergency minor	Six emergency minor
**Infections**	Two infections	One infection
**Quality of life**	Poor	Poor
**Survival**	20 years	1 year

Scenario 22

	Scenario A	Scenario B
**Planned major**	Two planned major	Two planned major
**Planned minor**	One planned minor	Two planned minor
**Emergency major**	One emergency major	One emergency major
**Emergency minor**	No emergency minor	Two emergency minor
**Infections**	Two infections	One infection
**Quality of life**	Poor	Poor
**Survival**	5 years	More than 20 years

Rivero-Arias O, *et al. Arch Dis Child* 2024; 109:377–386. doi: 10.1136/archdischild-2023-326156

Scenario 23

	Scenario A	Scenario B
**Planned major**	Six planned major	Six planned major
**Planned minor**	No planned minor	One planned minor
**Emergency major**	Six emergency major	Two emergency major
**Emergency minor**	Two emergency minor	Two emergency minor
**Infections**	No infections	Six infections
**Quality of life**	Fair	Good
**Survival**	5 years	5 years

Scenario 24

	Scenario A	Scenario B
**Planned major**	Two planned major	Two planned major
**Planned minor**	Two planned minor	Six planned minor
**Emergency major**	Two emergency major	Two emergency major
**Emergency minor**	Two emergency minor	Six emergency minor
**Infections**	Six infections	No infections
**Quality of life**	Poor	Poor
**Survival**	6 months	20 years

Scenario 25

	Scenario A	Scenario B
**Planned major**	Two planned major	Two planned major
**Planned minor**	No planned minor	No planned minor
**Emergency major**	One emergency major	Two emergency major
**Emergency minor**	One emergency minor	No emergency minor
**Infections**	Six infections	One infection
**Quality of life**	Good	Good
**Survival**	20 years	1 year

Scenario 26

	Scenario A	Scenario B
**Planned major**	One planned major	Two planned major
**Planned minor**	Six planned minor	Six planned minor
**Emergency major**	One emergency major	One emergency major
**Emergency minor**	Six emergency minor	One emergency minor
**Infections**	Two infections	No infections
**Quality of life**	Fair	Fair
**Survival**	20 years	5 years

Scenario 27

	Scenario A	Scenario B
**Planned major**	Two planned major	Six planned major
**Planned minor**	No planned minor	No planned minor
**Emergency major**	One emergency major	Six emergency major
**Emergency minor**	One emergency minor	No emergency minor
**Infections**	One infection	One infection
**Quality of life**	Fair	Fair
**Survival**	6 months	More than 20 years

Scenario 28

	Scenario A	Scenario B
**Planned major**	Six planned major	Six planned major
**Planned minor**	No planned minor	One planned minor
**Emergency major**	One emergency major	One emergency major
**Emergency minor**	No emergency minor	No emergency minor
**Infections**	No infections	One infection
**Quality of life**	Fair	Good
**Survival**	1 year	More than 20 years

Scenario 29

	Scenario A	Scenario B
**Planned major**	Two planned major	Six planned major
**Planned minor**	One planned minor	One planned minor
**Emergency major**	Two emergency major	Six emergency major

Scenario 30

	Scenario A	Scenario B
**Planned major**	Two planned major	Six planned major
**Planned minor**	No planned minor	One planned minor
**Emergency major**	Two emergency major	No emergency major

Rivero-Arias O, *et al. Arch Dis Child* 2024; 109:377–386. doi: 10.1136/archdischild-2023-326156

**Emergency minor**	One emergency minor	One emergency minor
**Infections**	Six infections	No infections
**Quality of life**	Poor	Fair
**Survival**	1 year	1 year

**Emergency minor**	Six emergency minor	Six emergency minor
**Infections**	Six infections	One infection
**Quality of life**	Fair	Fair
**Survival**	More than 20 years	More than 20 years

Scenario 31

	**Scenario A**	**Scenario B**
**Planned major**	Two planned major	Two planned major
**Planned minor**	No planned minor	Six planned minor
**Emergency major**	One emergency major	One emergency major
**Emergency minor**	Two emergency minor	No emergency minor
**Infections**	Two infections	One infection
**Quality of life**	Good	Poor
**Survival**	More than 20 years	More than 20 years

Scenario 32

	**Scenario A**	**Scenario B**
**Planned major**	Two planned major	Two planned major
**Planned minor**	No planned minor	No planned minor
**Emergency major**	Two emergency major	Two emergency major
**Emergency minor**	No emergency minor	Six emergency minor
**Infections**	Six infections	Two infections
**Quality of life**	Fair	Poor
**Survival**	5 years	20 years

Scenario 33

	**Scenario A**	**Scenario B**
**Planned major**	One planned major	One planned major
**Planned minor**	No planned minor	Two planned minor
**Emergency major**	Six emergency major	Six emergency major
**Emergency minor**	Two emergency minor	Two emergency minor
**Infections**	One infection	Six infections
**Quality of life**	Poor	Good
**Survival**	1 year	5 years

Scenario 34

	**Scenario A**	**Scenario B**
**Planned major**	No planned major	Six planned major
**Planned minor**	One planned minor	One planned minor
**Emergency major**	One emergency major	No emergency major
**Emergency minor**	No emergency minor	No emergency minor
**Infections**	Two infections	Six infections
**Quality of life**	Poor	Good
**Survival**	More than 20 years	More than 20 years

Scenario 35

	**Scenario A**	**Scenario B**
**Planned major**	No planned major	Six planned major
**Planned minor**	Six planned minor	No planned minor
**Emergency major**	One emergency major	No emergency major
**Emergency minor**	One emergency minor	One emergency minor
**Infections**	One infection	One infection
**Quality of life**	Good	Fair
**Survival**	More than 20 years	More than 20 years

Scenario 36

	**Scenario A**	**Scenario B**
**Planned major**	One planned major	One planned major
**Planned minor**	No planned minor	No planned minor
**Emergency major**	One emergency major	Two emergency major
**Emergency minor**	Six emergency minor	One emergency minor
**Infections**	No infections	Two infections
**Quality of life**	Poor	Good
**Survival**	More than 20 years	More than 20 years

Scenario 37

Scenario 38

	Scenario A	Scenario B
**Planned major**	One planned major	One planned major
**Planned minor**	No planned minor	Six planned minor
**Emergency major**	One emergency major	One emergency major
**Emergency minor**	No emergency minor	Two emergency minor
**Infections**	Two infections	One infection
**Quality of life**	Good	Good
**Survival**	1 year	20 years

	Scenario A	Scenario B
**Planned major**	One planned major	Six planned major
**Planned minor**	No planned minor	One planned minor
**Emergency major**	Six emergency major	Six emergency major
**Emergency minor**	Six emergency minor	Six emergency minor
**Infections**	Two infections	Two infections
**Quality of life**	Poor	Fair
**Survival**	1 year	20 years

Scenario 39

	Scenario A	Scenario B
**Planned major**	Two planned major	Two planned major
**Planned minor**	Six planned minor	Six planned minor
**Emergency major**	One emergency major	Two emergency major
**Emergency minor**	Two emergency minor	No emergency minor
**Infections**	No infections	One infection
**Quality of life**	Fair	Fair
**Survival**	6 months	5 years

Scenario 40

	Scenario A	Scenario B
**Planned major**	No planned major	Six planned major
**Planned minor**	No planned minor	No planned minor
**Emergency major**	No emergency major	No emergency major
**Emergency minor**	One emergency minor	No emergency minor
**Infections**	One infection	Six infections
**Quality of life**	Poor	Fair
**Survival**	More than 20 years	More than 20 years

Scenario 41

	Scenario A	Scenario B
**Planned major**	No planned major	Two planned major
**Planned minor**	One planned minor	Six planned minor
**Emergency major**	Six emergency major	Two emergency major
**Emergency minor**	Six emergency minor	Two emergency minor
**Infections**	Six infections	Six infections
**Quality of life**	Fair	Fair
**Survival**	More than 20 years	More than 20 years

Scenario 42

	Scenario A	Scenario B
**Planned major**	Two planned major	Two planned major
**Planned minor**	No planned minor	One planned minor
**Emergency major**	Six emergency major	Two emergency major
**Emergency minor**	Six emergency minor	Six emergency minor
**Infections**	Six infections	No infections
**Quality of life**	Poor	Poor
**Survival**	5 years	1 year

Scenario 43

	Scenario A	Scenario B
**Planned major**	One planned major	Two planned major
**Planned minor**	One planned minor	No planned minor
**Emergency major**	Two emergency major	Two emergency major
**Emergency minor**	Six emergency minor	No emergency minor
**Infections**	One infection	One infection
**Quality of life**	Poor	Poor
**Survival**	More than 20 years	1 year

Scenario 44

	Scenario A	Scenario B
**Planned major**	One planned major	Two planned major
**Planned minor**	Two planned minor	Two planned minor
**Emergency major**	Six emergency major	Six emergency major
**Emergency minor**	Two emergency minor	One emergency minor
**Infections**	Two infections	No infections
**Quality of life**	Good	Poor
**Survival**	More than 20 years	More than 20 years

Scenario 45

	Scenario A	Scenario B
**Planned major**	Two planned major	Two planned major
**Planned minor**	No planned minor	One planned minor
**Emergency major**	Six emergency major	Six emergency major
**Emergency minor**	Two emergency minor	One emergency minor
**Infections**	Two infections	Two infections
**Quality of life**	Fair	Good
**Survival**	More than 20 years	1 year

Scenario 46

	Scenario A	Scenario B
**Planned major**	Six planned major	No planned major
**Planned minor**	Two planned minor	No planned minor
**Emergency major**	Six emergency major	No emergency major
**Emergency minor**	Six emergency minor	No emergency minor
**Infections**	Six infections	No infections
**Quality of life**	Fair	Fair
**Survival**	Six months	One month

Scenario 47

	Scenario A	Scenario B
**Planned major**	Six planned major	No planned major
**Planned minor**	Six planned minor	No planned minor
**Emergency major**	Six emergency major	No emergency major
**Emergency minor**	Six emergency minor	No emergency minor
**Infections**	Six infections	No infections
**Quality of life**	Poor	Fair
**Survival**	One year	One month

Scenario 48

	Scenario A	Scenario B
**Planned major**	Six planned major	No planned major
**Planned minor**	Six planned minor	No planned minor
**Emergency major**	Six emergency major	No emergency major
**Emergency minor**	Six emergency minor	No emergency minor
**Infections**	Two infections	No infections
**Quality of life**	Poor	Fair
**Survival**	One year	One month

Scenario 49

	Scenario A	Scenario B
**Planned major**	Six planned major	No planned major
**Planned minor**	Six planned minor	No planned minor
**Emergency major**	Six emergency major	No emergency major
**Emergency minor**	Two emergency minor	No emergency minor
**Infections**	Six infections	No infections
**Quality of life**	Poor	Fair
**Survival**	One year	One month

Scenario 50

	Scenario A	Scenario B
**Planned major**	Six planned major	No planned major
**Planned minor**	Six planned minor	No planned minor
**Emergency major**	Two emergency major	No emergency major
**Emergency minor**	Six emergency minor	No emergency minor
**Infections**	Two infections	No infections
**Quality of life**	Poor	Fair
**Survival**	One year	One month

Scenario 51

	Scenario A	Scenario B
**Planned major**	Six planned major	No planned major
**Planned minor**	Two planned minor	No planned minor
**Emergency major**	Six emergency major	No emergency major

Scenario 52

	Scenario A	Scenario B
**Planned major**	Two planned major	No planned major
**Planned minor**	Six planned minor	No planned minor
**Emergency major**	Six emergency major	No emergency major

**Emergency minor**	Six emergency minor	No emergency minor
**Infections**	Two infections	No infections
**Quality of life**	Poor	Fair
**Survival**	6 months	One month

**Emergency minor**	Two emergency minor	No emergency minor
**Infections**	Six infections	No infections
**Quality of life**	Poor	Fair
**Survival**	6 months	One month

Scenario 53

	**Scenario A**	**Scenario B**
**Planned major**	Six planned major	No planned major
**Planned minor**	Two planned minor	No planned minor
**Emergency major**	Six emergency major	No emergency major
**Emergency minor**	Six emergency minor	No emergency minor
**Infections**	Six infections	No infections
**Quality of life**	Poor	Fair
**Survival**	6 months	One month

Scenario 54

	**Scenario A**	**Scenario B**
**Planned major**	Six planned major	No planned major
**Planned minor**	Six planned minor	No planned minor
**Emergency major**	Six emergency major	No emergency major
**Emergency minor**	Six emergency minor	No emergency minor
**Infections**	Six infections	No infections
**Quality of life**	Poor	Fair
**Survival**	6 months	One month

Scenario 55

	**Scenario A**	**Scenario B**
**Planned major**	Six planned major	No planned major
**Planned minor**	Six planned minor	No planned minor
**Emergency major**	Six emergency major	No emergency major
**Emergency minor**	Six emergency minor	No emergency minor
**Infections**	Two infections	No infections
**Quality of life**	Poor	Fair
**Survival**	20 years	One month

Scenario 56

	**Scenario A**	**Scenario B**
**Planned major**	Six planned major	No planned major
**Planned minor**	Two planned minor	No planned minor
**Emergency major**	Six emergency major	No emergency major
**Emergency minor**	Two emergency minor	No emergency minor
**Infections**	Six infections	No infections
**Quality of life**	Poor	Fair
**Survival**	5 years	One month

Scenario 57

	**Scenario A**	**Scenario B**
**Planned major**	Two planned major	No planned major
**Planned minor**	Six planned minor	No planned minor
**Emergency major**	Six emergency major	No emergency major
**Emergency minor**	Six emergency minor	No emergency minor
**Infections**	Six infections	No infections
**Quality of life**	Poor	Fair
**Survival**	6 months	One month

Scenario 58

	**Scenario A**	**Scenario B**
**Planned major**	Six planned major	No planned major
**Planned minor**	One planned minor	No planned minor
**Emergency major**	Six emergency major	No emergency major
**Emergency minor**	Six emergency minor	No emergency minor
**Infections**	Six infections	No infections
**Quality of life**	Poor	Fair
**Survival**	6 months	One month

Scenario 59

	**Scenario A**	**Scenario B**

Scenario 60

	**Scenario A**	**Scenario B**

**Planned major**	Six planned major	No planned major
**Planned minor**	Two planned minor	No planned minor
**Emergency major**	Six emergency major	No emergency major
**Emergency minor**	Six emergency minor	No emergency minor
**Infections**	Six infections	No infections
**Quality of life**	Poor	Fair
**Survival**	1 year	One month

**Planned major**	Six planned major	No planned major
**Planned minor**	Six planned minor	No planned minor
**Emergency major**	Two emergency major	No emergency major
**Emergency minor**	Six emergency minor	No emergency minor
**Infections**	Six infections	No infections
**Quality of life**	Poor	Fair
**Survival**	6 months	One month

Figure S3: Examples of CSOR Treatment Success Score (TSS)

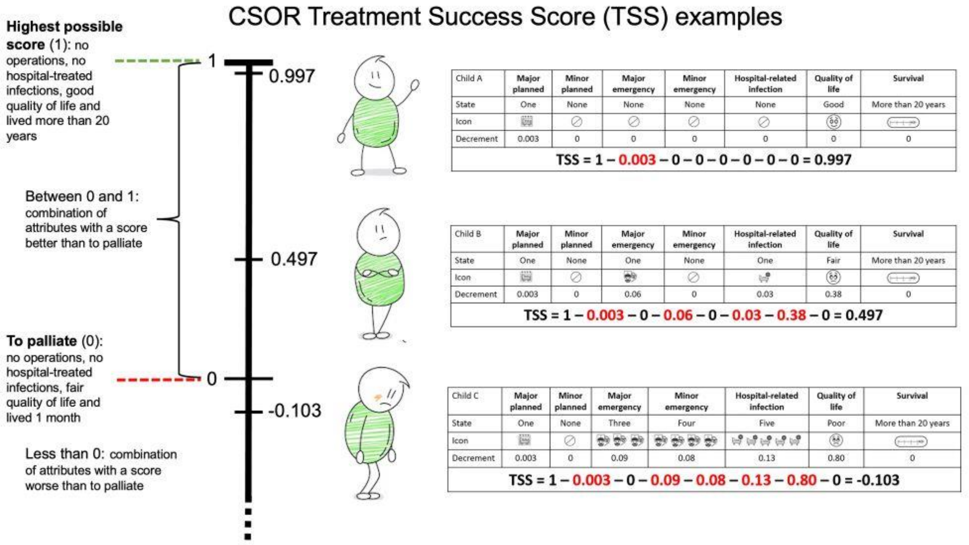

Table S1: Treated conditions of children and participants treated as a child completing the DCE exercise

	**Parent or carer of a child* (n = 147)**	**Participant treated as a child* (n = 106)**
Hirchsprung's	20 (13.6%)	1 (0.9%)
Necrotising enterocolitis	13 (8.8%)	0 (0%)
Gastroschisis	9 (6.1%)	3 (2.8%)
Oesophageal atresia	7 (4.8%)	1 (0.9%)
Congenital diaphragmatic hernia	5 (3.4%)	2 (1.9%)
Posterior urethral valves	1 (0.7%)	1 (0.9%)
Other surgical condition	96 (65.3%)	100 (94.3%)

*Some children and people treated reported having more than one treated condition

Rivero-Arias O, *et al. Arch Dis Child* 2024; 109:377–386. doi: 10.1136/archdischild-2023-326156

Table S2: Mean choice probabilities associated to each choice task by stakeholder group. Values indicate the probability of selecting scenario B

	**Parent or carer or treated as a child (n=253)**		**Healthcare professional (n=114)**		**General population (n=753)**	
Choice task	Probability of choosing B	Number of choices	Probability of choosing B	Number of choices	Probability of choosing B	Number of choices
1	0.520	50	0.517	29	0.538	143
2	0.203	64	0.167	24	0.269	156
3	0.125	64	0.292	24	0.237	156
4	0.929	56	0.810	21	0.859	142
5	0.320	50	0.172	29	0.350	143
6	0.732	56	0.524	21	0.746	142
7	0.120	50	0.103	29	0.147	143
8	0.900	50	0.966	29	0.832	143
9	0.694	62	0.926	27	0.796	157
10	0.050	60	0.038	26	0.058	155
11	0.268	56	0.571	21	0.303	142
12	0.516	62	0.296	27	0.427	157
13	0.906	64	1.000	24	0.897	156
14	0.891	64	1.000	24	0.929	156
15	0.700	50	0.448	29	0.636	143
16	0.320	50	0.517	29	0.406	143
17	0.758	62	0.630	27	0.720	157
18	0.940	50	0.897	29	0.881	143
19	0.120	50	0.103	29	0.126	143
20	0.054	56	0.095	21	0.021	142
21	0.150	60	0.154	26	0.116	155
22	0.883	60	0.731	26	0.884	155
23	0.952	62	1.000	27	0.828	157
24	0.828	64	0.667	24	0.840	156
25	0.054	56	0.095	21	0.042	142
26	0.145	62	0.185	27	0.197	157
27	0.855	62	0.963	27	0.885	157
28	1.000	56	1.000	21	0.965	142
29	0.867	60	0.846	26	0.748	155
30	0.839	56	0.952	21	0.732	142
31	0.000	60	0.038	26	0.045	155
32	0.468	62	0.444	27	0.605	157
33	0.929	56	1.000	21	0.958	142
34	0.969	64	0.958	24	0.853	156
35	0.109	64	0.042	24	0.109	156
36	0.969	64	0.958	24	0.923	156
37	0.983	60	0.962	26	0.916	155
38	0.935	62	1.000	27	0.924	157
39	0.920	50	0.931	29	0.902	143
40	0.817	60	0.923	26	0.723	155
41	0.887	62	0.963	27	0.752	157
42	0.375	56	0.429	21	0.380	142

43	0.150	60	0.192	26	0.155	155
44	0.031	64	0.125	24	0.051	156
45	0.117	60	0.154	26	0.116	155
46	0.842	19	1.000	12	0.547	53
47	0.727	22	0.857	7	0.722	54
48	0.810	21	1.000	8	0.840	50
49	0.828	29	0.909	11	0.774	53
50	0.722	18	1.000	8	0.764	55
51	1.000	13	1.000	7	0.872	47
52	0.800	20	1.000	6	0.765	51
53	0.824	17	0.818	11	0.872	47
54	0.462	13	0.917	12	0.756	45
55	0.300	20	0.833	12	0.385	52
56	0.650	20	1.000	5	0.446	56
57	0.958	24	0.714	7	0.771	48
58	0.583	24	0.857	7	0.953	43
59	0.800	15	1.000	5	0.811	53
60	0.765	17	0.889	9	0.891	46

Choice tasks 1-45 were obtained from the experimental design whereas choice tasks 46-60 were selected to understand the value of the palliative scenario by participants.

Table S3: Mixed multinomial logit model estimates of choice responses for stakeholders.

		Parent or carer or treated as a child (1)		Healthcare professional (2)		General population (3)		Palliative space scale (4)	
		Est	SE	Est	SE	Est	SE	Est	SE
**Planned Major**	One	-	-	-	-	-	-	-	-
	Two	-0.175	0.220	0.458	0.282	**-0.282**	**0.112**	0.003	0.023
	Six	-0.398	0.227	0.405	0.231	**-0.652**	**0.100**	-0.012	0.020
**Planned Minor**	One	-0.086	0.169	-	-	-0.145	0.101	-0.004	0.016
	Two	-	-	-	-	-	-	-	-
	Six	-0.197	0.185	-	-	-0.176	0.098	0.001	0.017
**Emergency Major**	One	-0.343	0.178	-0.425	0.275	-0.086	0.088	**-0.040**	**0.018**
	Two	**-0.533**	**0.263**	-0.079	0.294	-0.017	0.114	-0.035	0.022
	Six	**-1.293**	**0.364**	**-1.231**	**0.350**	**-0.819**	**0.124**	**-0.150**	**0.020**
**Emergency Minor**	One	-0.198	0.212	-	-	-0.010	0.090	-0.024	0.019
	Two	-	-	-	-	-	-	-	-
	Six	**-1.010**	**0.306**	-	-	**-0.496**	**0.101**	**-0.078**	**0.017**
**Hospital-treated Infection**	One	-	-	-	-	-	-	-	-
	Two	**-0.631**	**0.211**	-0.316	0.275	**-0.356**	**0.094**	**-0.073**	**0.018**
	Six	**-0.830**	**0.214**	**-0.667**	**0.274**	**-0.803**	**0.098**	**-0.103**	**0.018**
**Quality of Life**	Fair	**-2.273**	**0.328**	**-2.170**	**0.398**	**-1.486**	**0.139**	**-0.271**	**0.031**
	Poor	**-4.805**	**0.794**	**-4.769**	**0.761**	**-3.434**	**0.188**	**-0.580**	**0.039**
**Survival**	20 years	-0.258	0.347	-0.324	0.445	**-0.424**	**0.147**	-0.019	0.034
	5 years	**-2.121**	**0.467**	**-1.378**	**0.493**	**-1.991**	**0.170**	**-0.210**	**0.035**
	1 year	**-4.065**	**0.703**	**-3.486**	**0.492**	**-3.842**	**0.252**	**-0.466**	**0.037**
	6 months	**-4.825**	**0.834**	**-3.247**	**0.504**	**-4.173**	**0.293**	**-0.507**	**0.038**
**Palliative scenario**		**-9.134**	**1.665**	**-5.022**	**1.100**	**-7.124**	**0.702**		
**σ (Planned Major)**	One	-	-	-	-	-	-	-	-
	Two	0.730	0.473	**1.310**	**0.627**	**0.725**	**0.196**	**0.126**	**0.038**
	Six	0.581	0.528	0.016	0.022	0.206	0.563	-0.029	0.068
**σ (Planned Minor)**	One	-0.010	0.067	-	-	-0.021	0.033	-0.006	0.007
	Two	-	-	-	-	-	-	-	-
	Six	-0.049	0.112	-	-	0.110	0.206	0.006	0.010
**σ (Emergency Major)**	One	-0.194	0.261	-0.420	0.570	0.035	0.072	0.002	0.037
	Two	0.074	0.091	0.023	0.044	0.037	0.037	0.000	0.004
	Six	**1.307**	**0.358**	0.728	0.481	**0.635**	**0.190**	**-0.141**	**0.028**
**σ (Emergency Minor)**	One	0.734	0.486	-	-	0.304	0.520	0.088	0.056

	Two	-	-	-	-	-	-	-	-
	Six	-0.255	0.959	-	-	**0.930**	**0.171**	-0.009	0.053
**σ (Infection)**	One	-	-	-	-	-	-	-	-
	Two	0.141	0.096	-0.028	0.070	0.039	0.071	0.005	0.008
	Six	-0.622	0.421	0.608	0.475	0.002	0.098	0.062	0.043
**σ (Quality of life)**	Fair	0.121	0.138	-0.002	0.010	0.004	0.040	0.001	0.009
	Poor	**2.217**	**0.505**	**1.435**	**0.464**	**1.311**	**0.137**	**-0.234**	**0.028**
**σ (Survival)**	20 years	**1.691**	**0.622**	1.162	1.068	**0.938**	**0.305**	0.222	0.048
	5 years	0.673	0.939	-0.124	0.265	0.014	0.143	0.004	0.061
	1 year	0.901	0.570	0.794	0.545	**1.474**	**0.232**	**-0.081**	**0.038**
	6 months	**1.622**	**0.592**	**1.655**	**0.602**	**1.568**	**0.375**	**-0.172**	**0.040**
**σ (Palliative)**		**2.994**	**1.476**	-	-	2.184	1.302		
**Number of choices**		2,530		1,140		7,530		3,670	
**Number of participants**		253		114		753		367	
**Log-likelihood**		-1,044.09		-459.70		-3,382.888		-1527.84	

Bold estimates are statistically significant at 5%. Model estimated using maximum simulated likelihood in which normal distributions were specified for attributes; that is we estimated a mean (μ) and standard deviation (σ_θ^2), $iid \sim N(\mu, \sigma_\theta^2)$. Latent scale models (1), (2) and (3) were estimated using 5,000 Halton draws and the Broyden-Fletcher-Goldfarb-Shanno (BFGS) algorithm in Stata whereas model (4) was estimated in R using the Apollo package combining preferences from parents/carer/people treated and healthcare professionals using 5,000 Modified Latin Hypercube Sampling draws from a normal distribution.

Table S4: Mean scores by characteristics of infants with HD and gastroschisis who had quality of life data at 5-8 years of age

**Variables**	**n (%)**	**Mean (SD)**
Overall score	116	0.44 (0.28)
Surgical condition		
Gastroschisis	39	0.50 (0.20)
HD	77	0.41 (0.31)
Severe		
No	43	0.48 (0.25)
Yes	64	0.43 (0.29)
Missing	9	
Associated anomalies		
No	95	0.47 (0.26)
Yes	19	0.32 (0.34)
Missing	2	
Sex		
Female	77	0.44 (0.27)
Male	39	0.43 (0.29)
Birthweight, grams		
≥2500	85	0.42 (0.29)
<2500	28	0.50 (0.23)
Missing	3	
Gestational age, weeks		
28 – <32	5	0.21 (0.35)
32 – <37	21	0.46 (0.19)
≥37	89	0.45 (0.29)
Missing	1	
Ethnicity		
White	102	0.45 (0.28)
Black	3	0.43 (0.23)
Asian	5	0.29 (0.26)
Mixed/Other	4	0.47 (0.45)
Missing	2	
Maternal age, years		
<20	10	0.46 (0.27)
20 – <25	26	0.49 (0.19)
25 – <30	20	0.45 (0.30)
30 – <35	25	0.51 (0.27)
≥35	24	0.36 (0.28)
Missing	11	

Note: No infants in the gestational age <28 weeks category.

Table S5: Mean scores by characteristics of infants across the full sample with partial CSOR TSSs (excluding quality of life)

Variables	n	Mean (SD)
Overall score	1,383	0.91 (0.23)
Surgical condition		
CDH	212	0.79 (0.39)
Gastroschisis	353	0.96 (0.13)
HD	305	0.95 (0.14)
NEC	243	0.77 (0.36)
OA	151	0.94 (0.20)
PUV	119	0.98 (0.07)
Associated anomalies		
No	1,054	0.91 (0.24)
Yes	319	0.85 (0.31)
Missing	10	
Sex		
Female	716	0.91 (0.24)
Male	663	0.88 (0.28)
Missing	4	
Birthweight, grams		
≥2500	754	0.93 (0.22)
<2500	585	0.85 (0.30)
Missing	44	
Gestational age		
<28	143	0.75 (0.36)
28 – <32	91	0.78 (0.36)
32 – <37	305	0.89 (0.26)
≥37	824	0.94 (0.21)
Missing	20	
Ethnicity		
White	1,121	0.91 (0.25)
Black	58	0.78 (0.35)
Asian	103	0.90 (0.26)
Mixed/Other	58	0.88 (0.28)
Missing	43	
Maternal age, years		
<20	151	0.94 (0.18)
20 – <25	288	0.90 (0.26)
25 – <30	283	0.89 (0.27)
30 – <35	275	0.91 (0.25)
≥35	221	0.83 (0.33)
Missing	165	

Note: 140/143 infants who were less than 28 weeks of gestation at birth had NEC.

References:

1. Sherratt FC, Allin BSR, Kirkham JJ, et al. Core outcome set for uncomplicated acute appendicitis in children and young people. *Br J Surg* 2020;107(8):1013-22. doi: 10.1002/bjs.11508 [published Online First: 2020/03/18]
2. Allin BSR, Bradnock T, Kenny S, et al. NETS(1HD) study: development of a Hirschsprung's disease core outcome set. *Arch Dis Child* 2017;102(12):1143-51. doi: 10.1136/archdischild-2017-312901 [published Online First: 20170807]
3. Allin BSR, Hall NJ, Ross AR, et al. Development of a gastroschisis core outcome set. *Archives of disease in childhood Fetal and neonatal edition* 2019;104(1):F76-F82. doi: 10.1136/archdischild-2017-314560 [published Online First: 20180314]
4. Webbe JWH, Duffy JMN, Afonso E, et al. Core outcomes in neonatology: development of a core outcome set for neonatal research. *Archives of disease in childhood Fetal and neonatal edition* 2020;105(4):425-31. doi: 10.1136/archdischild-2019-317501 [published Online First: 20191115]
5. Rivero-Arias O, Buckell J, Allin B, et al. Using stated-preferences methods to develop a summary metric to determine successful treatment of children with a surgical condition: a study protocol. *BMJ Open* 2022;12(6):e062833. doi: 10.1136/bmjopen-2022-062833 [published Online First: 20220609]
6. ChoiceMetrics. Ngene 1.2 User Manual & Reference Guide. Australia2018.
7. Vass CM, Davison NJ, Vander Stichele G, et al. A Picture is Worth a Thousand Words: The Role of Survey Training Materials in Stated-Preference Studies. *The patient* 2020;13(2):163-73. doi: 10.1007/s40271-019-00391-w
8. Train K, Weeks M. Discrete Choice Models in Preference Space and Willingness-to-Pay Space. In: Scarpa R, Alberini A, eds. Applications of Simulation Methods in Environmental and Resource Economics. Dordrecht: Springer Netherlands 2005:1-16.

Rivero-Arias O, *et al. Arch Dis Child* 2024; 109:377–386. doi: 10.1136/archdischild-2023-326156
